# Supplementary material for: Integrated Analysis of the Intestinal Microbiota and Transcriptome of Fenneropenaeus chinensis Response to Low-Salinity Stress
Source: Biology (Basel). 2023 Dec 7;12(12):1502. doi: 10.3390/biology12121502 (PMC10741032; doi:10.3390/biology12121502)
Supplement: Supplementary file 1 [file biology-12-01502-s001.zip › biology-2656527-supplementary.pdf]

Integrated Analysis of the Intestinal Microbiota and Transcriptome of *Fenneropenaeus chinensis* Response to Low-Salinity Stress

Caijuan Tian [1,2], Qiong Wang [2,3], Jiajia Wang [2,3], Jitao Li [2,3], Chenhui Guan [2,4], Yuying He [2,3,*] and Huan Gao [1,*]

[1] Jiangsu Key Laboratory of Marine Bioresources and Environment / Jiangsu Key Laboratory of Marine Biotechnology, Jiangsu Ocean University, Lianyungang 222005, China; tcaijuan@163.com

[2] National Key Laboratory of Mariculture Biobreeding and Sustainable Goods, Yellow Sea Fisheries Research Institute, Chinese Academy of Fishery Sciences, Qingdao 266071, China; wangqiong@ysfri.ac.cn (Q.W.); wangjj@ysfri.ac.cn (J.W.); lijt@ysfri.ac.cn (J.L.); guanchenhui678@163.com (C.G.)

[3] Function Laboratory for Marine Fisheries Science and Food Production Processes, Pilot National Laboratory for Marine Science and Technology, Qingdao 266200, China

[4] School of Marine Science and Engineering, Qingdao Agricultural University, Qingdao, 266237, China

* Correspondence: heyy@ysfri.ac.cn (Y.H.); huanmr@163.com (H.G.); Tel.: +86-13780639650 (Y.H.); +86-13951258651 (H.G.)

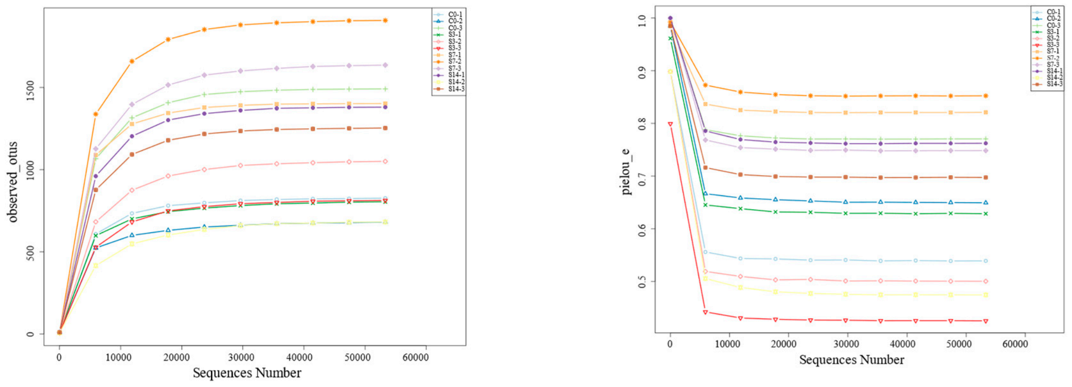

Figure S1. Rarefaction curve analysis of intestinal microbes. the index of observed_otus and pielou_e.

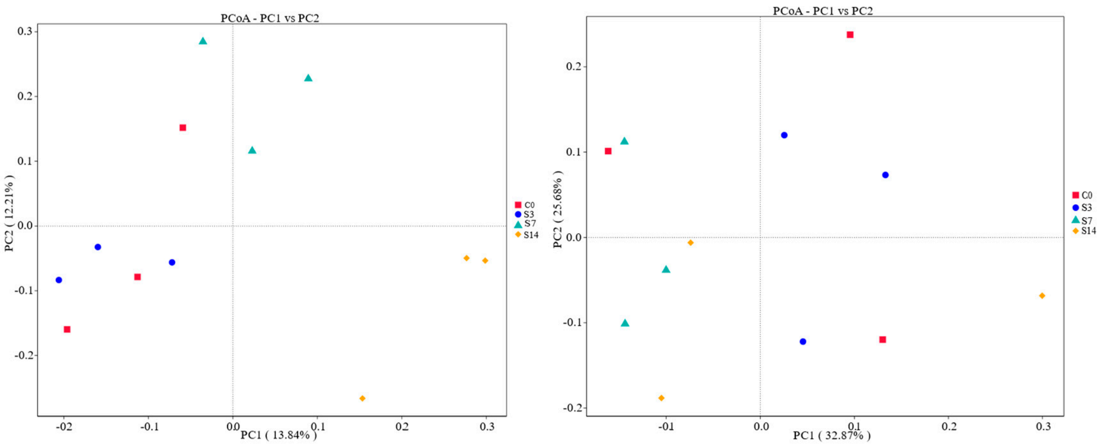

Figure S2. Beta diversity indicated by PCoA based on unweighted and weighted UniFrac distances

Table S1. Statistic of the 16S amplicon sequencing data.

Sample_name	Total_reads	Combined_reads	Uncombined_reads	Percent_combined(%)	Combined_base(bp)	Min_len(bp)	Max_len(bp)	Avg_len(bp)
C0_1	87,398	59,224	28,174	67.76	14,893,986	43	389	251
C0_1	80,922	66,226	14,696	81.84	16,748,030	43	388	253
C0_1	84,491	64,354	20,137	76.17	16,180,222	43	390	251
S3_1	87,441	71,544	15,897	81.82	18,056,773	43	390	252
S3_2	79,421	76,798	2,623	96.7	19,383,148	44	389	252
S3_3	78,920	68,625	10,295	86.96	17,291,655	39	390	252
S7_1	79,163	77,201	1,962	97.52	19,724,788	44	389	255
S7_2	80,870	78,563	2,307	97.15	20,176,572	15	390	257
S7_3	79,866	73,137	6,729	91.57	18,714,543	19	390	256
S14_1	82,712	81,383	1,329	98.39	20,948,076	15	390	257
S14_2	90,777	88,593	2,184	97.59	22,543,960	44	389	254
S14_3	80,582	80,017	565	99.3	20,666,293	24	390	258
#Total	992,563	885,665	106,898	89.23	225,328,046	44	388	254

Table S2 Alpha diversity of intestinal microbes in *F. chinensis* exposed low-salinity stress.

Group_Name	chao1	dominance	observed_otus	shannon	simpson
C0	1002.98±429.60^{b}	0.09±0.08^{ab}	999±432.29^{bc}	6.48±1.49^{ab}	0.91±0.08^{ab}
S3	892.94±139.83^{c}	0.22±0.12^{a}	888.67±139.78^{c}	5.06±0.98^{b}	0.78±0.12^{bc}
S7	1652.41±254.48^{a}	0.03±0.01^{b}	1648.67±253.24^{a}	8.62±0.65^{a}	0.99±0.01^{a}
S14	1107.22±372.96^{ab}	0.07±0.06^{b}	1104.67±372.36^{a}	6.53±1.83^{a}	0.93±0.06^{a}

Table S3. (a). The top 10 abundant microflora composition at phylum level in the intestine of *F. chinensis*. (b) The top 30 abundant microflora composition at genus level in the intestine of *F.chinensis*.

(a)

Taxonomy	C0	S3	S7	S14
Proteobacteria	0.54029	0.705284	0.380555	0.514228
Firmicutes	0.181264	0.095556	0.168865	0.311617
Actinobacteriota	0.046559	0.024617	0.079586	0.015901
Bacteroidota	0.04035	0.049185	0.083982	0.045052
Cyanobacteria	0.047822	0.003527	0.038099	0.014894
Acidobacteriota	0.009442	0.008085	0.055075	0.010442
Chloroflexi	0.003245	0.001238	0.013806	0.002701
Gemmatimonadota	0.003683	0.003533	0.012862	0.00484
Myxococcota	0.002126	0.002001	0.011061	0.002682
Verrucomicrobiota	0.003645	0.001232	0.00821	0.004039
Others	0.10638	0.096719	0.11706	0.059321

(b)

Taxonomy	C0	S3	S7	S14
Photobacterium	0.118498	0.223921	0.026456	0.000538
Sphingomonas	0.006353	0.161574	0.019953	0.001432
Vibrio	0.182715	0.133492	0.054131	0.064523
Shewanella	0.000713	0.000682	0.00539	0.112695
Pseudomonas	0.016282	0.015713	0.036498	0.113827
Chloroplast	0.045858	0.003095	0.037536	0.014257
Lactobacillus	0.017771	0.010142	0.031158	0.062847
Ralstonia	0.004758	0.007122	0.007378	0.045439
Colwellia	0	0	0.000175	0.02463
Cohaesibacter	0.000044	0	0	0.023942
Fusibacter	0.000169	0	0	0.023354
Muribaculaceae	0.00514	0.019278	0.020209	0.00604
Lachnospiraceae_NK4A136_group	0.001101	0.003302	0.002801	0.026687
Delftia	0.001601	0.017177	0.002045	0.001632
Escherichia-Shigella	0.013068	0.030808	0.009179	0.004202
Lactococcus	0.000675	0.000294	0.000388	0.012806
Collinsella	0.011186	0.001263	0.001025	0.000613
Bacteroides	0.012287	0.013187	0.013781	0.013756
Halomonas	0.014732	0.003402	0.002851	0.000607
Blautia	0.01108	0.006078	0.007128	0.003983
Vicinamibacteraceae	0.001901	0.001157	0.011749	0.003108
Ensifer	0.000363	0.008885	0.0004	0.000163
Parabacteroides	0.00362	0.001895	0.01083	0.001357
Enterococcus	0.003802	0.001432	0.009811	0.00272
RB41	0.000863	0.000625	0.008122	0.000531
Peptoclostridium	0.006703	0.000481	0.000644	0.000106
Dietzia	0.006859	0.002051	0.001438	0.00005
Streptococcus	0.005296	0.004021	0.010936	0.00192
Staphylococcus	0.002895	0.006559	0.007216	0.001369

Mesorhizobium	0.000306	0.000394	0.005209	0
Peptoclostridium	0.006703	0.000481	0.000644	0.000106
Others	0.278783	0.1776	0.315619	0.245093

Table S4. Statistic of RNA-Seq sequencing data of the *F. chinensis* intestine.

sample	library	raw_reads	raw_bases	clean_reads	clean_bases	error_rate	Q20	Q30	GC_pct
S14_1	FRAS220214198-1r	42331656	6.35G	40598134	6.09G	0.03	96.24	90.54	45.33
S3_3	FRAS220214194-1r	47050152	7.06G	45075000	6.76G	0.03	96.1	90.28	45.1
C0_1	FRAS220214189-1r	41007934	6.15G	39726002	5.96G	0.03	96.49	90.99	42.78
C0_3	FRAS220214191-1r	40857542	6.13G	39703800	5.96G	0.03	96.53	91.08	43.55
C0_2	FRAS220214190-1r	41197662	6.18G	40280688	6.04G	0.03	96.26	90.51	41.35
S7_2	FRAS220214196-1r	47597628	7.14G	46105076	6.92G	0.03	95.99	89.99	44.39
S7_3	FRAS220214197-1r	46914554	7.04G	45618852	6.84G	0.03	96.5	90.99	44.66
S14_3	FRAS220214200-1r	42280120	6.34G	41055098	6.16G	0.03	96.57	91.15	41.69
S7_1	FRAS220214195-1r	49828426	7.47G	47951552	7.19G	0.03	96.63	91.31	44.88
S3_1	FRAS220214192-1r	44885344	6.73G	42939274	6.44G	0.03	96.15	90.29	41.46
S14_2	FRAS220214199-1r	42728448	6.41G	40850776	6.13G	0.03	96.42	90.86	42.89
S3_2	FRAS220214193-1b	46005990	6.9G	44261912	6.64G	0.03	96.8	91.63	46.16

Table S5. The mapping result of the RNA-seq data.

sample	total_reads	total_map	unique_map	multi_map	read1_map	read2_map	positive_map	negative_map	splice_map	unsplice_map	proper_map
S14_1	40598134	36515302(89.94%)	34928994(86.04%)	1586308(3.91%)	17674715(43.54%)	17254279(42.5%)	17466111(43.02%)	17462883(43.01%)	12965230(31.94%)	21963764(54.1%)	32681334(80.5%)
S3_3	45075000	39637193(87.94%)	38646590(85.74%)	990603(2.2%)	19590915(43.46%)	19055675(42.28%)	19341092(42.91%)	19305498(42.83%)	14183063(31.47%)	24463527(54.27%)	35312786(78.34%)
C	397	35507	34250	12568	17278	16972	17165	17085	11054	23196	31912

0_1	26002	768(89.38%)	921(86.22%)	47(3.16%)	351(43.49%)	570(42.72%)	768(43.21%)	153(43.01%)	180(27.83%)	741(58.39%)	448(80.33%)
C0_3	39703800	35913683(90.45%)	34961124(88.05%)	952559(2.4%)	17627692(44.4%)	17333432(43.66%)	17496994(44.07%)	17464130(43.99%)	12303348(30.99%)	22657776(57.07%)	32944598(82.98%)
C0_2	40280688	36642687(90.97%)	34949655(86.77%)	1693032(4.2%)	17680543(43.89%)	17269112(42.87%)	17465104(43.36%)	17484551(43.41%)	9398323(23.33%)	25551332(63.43%)	33043218(82.03%)
S7_2	46105076	41533256(90.08%)	39509283(85.69%)	2023973(4.39%)	20066056(43.52%)	19443227(42.17%)	19745559(42.83%)	19763724(42.87%)	13995702(30.36%)	25513581(55.34%)	36504494(79.18%)
S7_3	45618852	39791109(87.23%)	38106303(83.53%)	1684806(3.69%)	19246675(42.19%)	18859628(41.34%)	19131239(41.94%)	18975064(41.59%)	12723780(27.89%)	25382523(55.64%)	34464914(75.55%)
S14_3	41055098	37407651(91.12%)	35920851(87.49%)	1486800(3.62%)	18098331(44.08%)	17822520(43.41%)	17941711(43.7%)	17979140(43.79%)	10427251(25.4%)	25493600(62.1%)	34065036(82.97%)
S7_1	47951552	43336362(90.38%)	41455766(86.45%)	1880596(3.92%)	20911115(43.61%)	20544651(42.84%)	20748024(43.27%)	20707742(43.18%)	15165151(31.63%)	26290615(54.83%)	38674032(80.65%)
S3_1	42939274	36600784(85.24%)	35991904(83.82%)	608880(1.42%)	18213424(42.42%)	17778480(41.4%)	17988983(41.89%)	18002921(41.93%)	9957289(23.19%)	26034615(60.63%)	34105572(79.43%)
S14_2	40850776	36634452(89.68%)	35176458(86.11%)	1457994(3.57%)	17755672(43.46%)	17420786(42.64%)	17587180(43.05%)	17589278(43.06%)	11250242(27.54%)	23926216(58.57%)	32252928(78.95%)
S3_2	44261912	38754612(87.56%)	37428916(84.56%)	1325696(3.0%)	18907979(42.72%)	18520937(41.84%)	18745129(42.35%)	18683787(42.21%)	14661631(33.12%)	22767285(51.44%)	34643964(78.27%)

Table S6. The premer sequences of genes used for RT-qPCR.

Primers	Primers information	Sequences(5′-3′)	Purpose
ARSI-F1	arylsulfatase I	TGCTCGTGCCAGGAACATCATAAG	RT-qPCR
ARSI-R1		CGTGGACCGATTGTAGGGAAAGC	RT-qPCR
LAT-2-F2	latrophilin-like protein LAT-2	TGAGCATCAGCAGCGACAACTTC	RT-qPCR
LAT-2-R2		CTCGTAGCCCTGACCCTCCATAC	RT-qPCR
LGMN-F3	legumain	ACCTCGGTGTCTACGCTGTCTC	RT-qPCR
LGMN-		CCCTGTCCGTGTCCTCCATCC	RT-

R3			qPCR
PRCP-F4	lysosomal Pro-X carboxypeptidase	GGAGGAGTCATGTGGAATGTGAGTG	RT-qPCR
PRCP-R4		GGTCATTGGGATTGGAGCCTCTAAG	RT-qPCR
MFAP4-F5	Microfibrillar-associated protein 4	GCTTGACCGCCACGCACTAC	RT-qPCR
MFAP4-R5		ACCAGCCGCCCTCTTTCTCG	RT-qPCR
ProCTSL-F6	procathepsin L-like	TGACGATGGCACAGATTACTGGTTG	RT-qPCR
ProCTSL-R6		AGGAGGCGGAGGAGGCAATG	RT-qPCR
H1.2-F7	histone H1.2	GAGGAGGAAGAGGAGGAGGAAGAAG	RT-qPCR
H1.2-R7		GTTTGGACTTGGCTTTGGGCTTTAC	RT-qPCR
MFE-F8	methyl farnesoate epoxidase	GCAAATGACGACTTAAACGGACGAC	RT-qPCR
MFE-R8		CCTTCCACGAACATCACGCCTAG	RT-qPCR
PTER-F9	phosphotriesterase-related protein	TACTATCCAGGCAGCGGCTCATG	RT-qPCR
PTER-R9		ACCATCTTCTTCACATCACCACCAG	RT-qPCR
CPB-1-F10	cytoplasmic polyadenylation element-binding protein 1	AGAATGGTCAGACTGGCAATGCTG	RT-qPCR
CPB-1-R10		AGTGCGAGGAGTGAGTGGAGTG	RT-qPCR
β-actin	β-actin	AGTAGCCGCCCTGGTTGTAGA	RT-qPCR
β-actin		TTCTCCATGTCGTCCCAGT	RT-qPCR

Table S7. (a) Gene ontology (GO) terms of the DEGs in S3 vs. C0 group. (b) Gene ontology (GO) terms of the DEGs in S7 vs. C0 group. (c) Gene ontology (GO) terms of the DEGs in S14 vs. C0 group.

(a)

Category	GOID	Description	GeneRatio	BgRatio	pvalue	padj	Count	Up	Down
BP	GO:0006508	proteolysis	62/330	393/3632	4.08E-06	0.000941	62	14	48
BP	GO:0006	protein	17/330	74/363	0.0002	0.0109	17	0	17

	486	glycosylation		2	4	68			
BP	GO:0009101	glycoprotein biosynthetic process	17/330	74/3632	0.00024	0.010968	17	0	17
BP	GO:0043413	macromolecule glycosylation	17/330	74/3632	0.00024	0.010968	17	0	17
BP	GO:0070085	glycosylation	17/330	74/3632	0.00024	0.010968	17	0	17
BP	GO:0009100	glycoprotein metabolic process	17/330	75/3632	0.000285	0.010968	17	0	17
BP	GO:0016051	carbohydrate biosynthetic process	5/330	11/3632	0.001751	0.050354	5	1	4
BP	GO:1901137	carbohydrate derivative biosynthetic process	21/330	118/3632	0.001799	0.050354	21	1	20
BP	GO:0006979	response to oxidative stress	9/330	33/3632	0.002039	0.050354	9	0	9
BP	GO:0007156	homophilic cell adhesion via plasma membrane adhesion molecules	8/330	28/3632	0.002616	0.050354	8	1	7

CC	GO:0005576	extracellular region	38/212	176/1947	1.04E-05	0.000458	38	4	34
CC	GO:0005615	extracellular space	8/212	19/1947	0.000452	0.009954	8	1	7
CC	GO:0044421	extracellular region part	8/212	23/1947	0.001992	0.029213	8	1	7
CC	GO:0098791	Golgi subcompartment	5/212	12/1947	0.006089	0.066982	5	1	4
CC	GO:0005794	Golgi apparatus	6/212	22/1947	0.025936	0.190194	6	1	5
CC	GO:0044431	Golgi apparatus part	6/212	22/1947	0.025936	0.190194	6	1	5
CC	GO:0031984	organelle subcompartment	6/212	39/1947	0.245422	1	6	1	5
CC	GO:0016459	myosin complex	4/212	28/1947	0.363996	1	4	1	3
CC	GO:0012505	endomembrane system	10/212	83/1947	0.417443	1	10	2	8
CC	GO:0044459	plasma membrane part	2/212	17/1947	0.567465	1	2	2	0
MF	GO:0008146	sulfotransferase activity	34/548	125/6113	1.76E-09	3.07E-07	34	0	34
MF	GO:0016782	transferase activity, transferring sulfur-containing	34/548	130/6113	5.37E-09	4.67E-07	34	0	34

		groups							
MF	GO:0004175	endopeptidase activity	49/548	248/6113	6E-08	3.48E-06	49	8	41
MF	GO:0008233	peptidase activity	66/548	388/6113	1.33E-07	5.79E-06	66	14	52
MF	GO:0008236	serine-type peptidase activity	39/548	185/6113	2.48E-07	6.33E-06	39	7	32
MF	GO:0017171	serine hydrolase activity	39/548	185/6113	2.48E-07	6.33E-06	39	7	32
MF	GO:0004252	serine-type endopeptidase activity	37/548	171/6113	2.55E-07	6.33E-06	37	5	32
MF	GO:0070011	peptidase activity, acting on L-amino acid peptides	64/548	383/6113	4.1E-07	8.93E-06	64	12	52
MF	GO:0008417	fucosyltransferase activity	11/548	29/6113	2.1E-05	0.000406	11	0	11
MF	GO:0008061	chitin binding	25/548	121/6113	5.27E-05	0.000917	25	2	23

(b)

Category	GOID	Description	GeneRatio	BgRatio	pvalue	padj	Count	Up	Down
BP	GO:0032501	multicellular organismal process	5/90	40/3602	0.002874	0.221889	5	4	1
BP	GO:0030029	actin filament-based process	3/90	14/3602	0.004498	0.221889	3	0	3
BP	GO:0030036	actin cytoskeleton organization	3/90	14/3602	0.004498	0.221889	3	0	3
BP	GO:0034622	cellular protein-containing complex assembly	4/90	41/3602	0.018239	0.366098	4	0	4

BP	GO:0007275	multicellular organism development	3/90	25/3602	0.023319	0.366098	3	2	1
BP	GO:0055085	transmembrane transport	17/90	412/3602	0.024064	0.366098	17	11	6
BP	GO:0007015	actin filament organization	2/90	10/3602	0.024388	0.366098	2	0	2
BP	GO:0007010	cytoskeleton organization	3/90	27/3602	0.028616	0.366098	3	0	3
BP	GO:0097435	supramolecular fiber organization	2/90	11/3602	0.029329	0.366098	2	0	2
BP	GO:0032502	developmental process	3/90	28/3602	0.031484	0.366098	3	2	1
CC	GO:0000786	nucleosome	1/56	10/2000	0.247719	0.995608	1	0	1
CC	GO:0000428	DNA-directed RNA polymerase complex	1/56	11/2000	0.268888	0.995608	1	0	1
CC	GO:0000785	chromatin	1/56	11/2000	0.268888	0.995608	1	0	1
CC	GO:0033177	proton-transporting two-sector ATPase complex, proton-transporting domain	1/56	11/2000	0.268888	0.995608	1	0	1
CC	GO:0044815	DNA packaging complex	1/56	11/2000	0.268888	0.995608	1	0	1
CC	GO:0055029	nuclear DNA-directed RNA polymerase complex	1/56	11/2000	0.268888	0.995608	1	0	1
CC	GO:0032993	protein-DNA complex	1/56	12/2000	0.289473	0.995608	1	0	1
CC	GO:0015629	actin cytoskeleton	2/56	39/2000	0.298725	0.995608	2	1	1
CC	GO:0030880	RNA polymerase complex	1/56	13/2000	0.309488	0.995608	1	0	1
CC	GO:0031410	cytoplasmic vesicle	1/56	13/2000	0.309488	0.995608	1	1	0
MF	GO:0008484	sulfuric ester hydrolase activity	3/140	12/6327	0.002015	0.177779	3	2	1
MF	GO:0043565	sequence-specific DNA binding	7/140	86/6327	0.0028	0.177779	7	6	1
MF	GO:0016705	oxidoreductase activity, acting on paired donors, with incorporation or	6/140	81/6327	0.008787	0.371998	6	3	3

		reduction of molecular oxygen							
MF	GO:0003700	DNA-binding transcription factor activity	7/140	114/6327	0.012876	0.39257	7	7	0
MF	GO:0004807	triose-phosphate isomerase activity	2/140	10/6327	0.019473	0.39257	2	2	0
MF	GO:0004806	triglyceride lipase activity	2/140	11/6327	0.023459	0.39257	2	2	0
MF	GO:0008194	UDP-glycosyltransferase activity	4/140	53/6327	0.029135	0.39257	4	2	2
MF	GO:0016861	intramolecular oxidoreductase activity, interconverting aldoses and ketoses	2/140	13/6327	0.032324	0.39257	2	2	0
MF	GO:0005506	iron ion binding	5/140	81/6327	0.0331	0.39257	5	2	3
MF	GO:0140110	transcription regulator activity	7/140	139/6327	0.033995	0.39257	7	7	0

(c)

Category	GOID	Description	GeneRatio	BgRatio	pvalue	padj	Count	Up	Down
BP	GO:0007015	actin filament organization	4/171	10/3638	0.000792	0.080942	4	0	4
BP	GO:0097435	supramolecular fiber organization	4/171	11/3638	0.0012	0.080942	4	0	4
BP	GO:0006629	lipid metabolic process	13/171	107/3638	0.001306	0.080942	13	7	6
BP	GO:0033043	regulation of organelle organization	4/171	13/3638	0.002413	0.086523	4	0	4
BP	GO:0030029	actin filament-based process	4/171	14/3638	0.003256	0.086523	4	0	4
BP	GO:0030036	actin cytoskeleton organization	4/171	14/3638	0.003256	0.086523	4	0	4
BP	GO:0051128	regulation of cellular component organization	4/171	14/3638	0.003256	0.086523	4	0	4
BP	GO:0006	anion	5/171	27/363	0.0075	0.1554	5	3	2

	820	transport		8	2	07			
BP	GO:0007010	cytoskeleton organization	5/171	27/3638	0.00752	0.155407	5	0	5
BP	GO:0006631	fatty acid metabolic process	3/171	10/3638	0.009589	0.17835	3	1	2
CC	GO:0015629	actin cytoskeleton	6/81	39/1946	0.004681	0.215305	6	0	6
CC	GO:0044430	cytoskeletal part	6/81	54/1946	0.022532	0.474535	6	0	6
CC	GO:0005856	cytoskeleton	6/81	58/1946	0.030948	0.474535	6	0	6
CC	GO:0005576	extracellular region	12/81	181/1946	0.067023	0.770763	12	7	5
CC	GO:0000786	nucleosome	1/81	10/1946	0.346986	0.999259	1	0	1
CC	GO:0000785	chromatin	1/81	11/1946	0.374308	0.999259	1	0	1
CC	GO:0005777	peroxisome	1/81	11/1946	0.374308	0.999259	1	0	1
CC	GO:0042579	microbody	1/81	11/1946	0.374308	0.999259	1	0	1
CC	GO:0044815	DNA packaging complex	1/81	11/1946	0.374308	0.999259	1	0	1
CC	GO:0032993	protein-DNA complex	1/81	12/1946	0.400499	0.999259	1	0	1
MF	GO:0008061	chitin binding	12/270	128/6132	0.010368	0.825828	12	6	6
MF	GO:0008146	sulfotransferase activity	12/270	131/6132	0.012342	0.825828	12	2	10
MF	GO:0016782	transferase activity, transferring sulfur-containing groups	12/270	136/6132	0.016257	0.825828	12	2	10
MF	GO:0008509	anion transmembrane transporter activity	3/270	14/6132	0.02142	0.825828	3	2	1
MF	GO:0003779	actin binding	4/270	31/6132	0.045419	0.825828	4	0	4
MF	GO:0016	oxidoreductas	24/270	384/61	0.0503	0.8258	24	8	16

	491	e activity		32	94	28			
MF	GO:0016702	oxidoreductase activity, acting on single donors with incorporation of molecular oxygen, incorporation of two atoms of oxygen	3/270	20/6132	0.0553	0.825828	3	1	2
MF	GO:0008233	peptidase activity	25/270	409/6132	0.0576	0.825828	25	17	8
MF	GO:0008234	cysteine-type peptidase activity	6/270	63/6132	0.057619	0.825828	6	5	1
MF	GO:0016627	oxidoreductase activity, acting on the CH-CH group of donors	4/270	34/6132	0.060488	0.825828	4	0	4

Table S8. (a) The KEGG pathways enriched by the DEGs in S3 vs. C0 group. (b) The KEGG pathways enriched by the DEGs in S7 vs. C0 group. (c) The KEGG pathways enriched by the DEGs in S14 vs. C0 group.

(a)

KEGGID	Description	GeneRatio	pvalue	Count	Up	Down
dme00513	Various types of N-glycan biosynthesis	18/173	5.40E-08	18	1	17
dme00520	Amino sugar and nucleotide sugar metabolism	13/173	5.41E-05	13	3	10
dme00051	Fructose and mannose metabolism	9/173	0.000344584	9	4	5
dme00532	Glycosaminoglycan biosynthesis - chondroitin sulfate / dermatan sulfate	6/173	0.002045838	6	0	6
dme00512	Mucin type O-glycan biosynthesis	6/173	0.002591281	6	0	6
dme00531	Glycosaminoglycan degradation	6/173	0.00400061	6	2	4
dme00250	Alanine, aspartate and glutamate metabolism	7/173	0.005281357	7	0	7
dme00500	Starch and sucrose metabolism	6/173	0.005910002	6	0	6
dme00350	Tyrosine metabolism	5/173	0.005957441	5	0	5
dme04213	Longevity regulating pathway - multiple species	9/173	0.010810364	9	2	7
dme00603	Glycosphingolipid biosynthesis - globo and isoglobo series	4/173	0.017296309	4	0	4
dme00360	Phenylalanine metabolism	3/173	0.019729117	3	0	3
dme00514	Other types of O-glycan biosynthesis	7/173	0.02254687	7	0	7
dme00604	Glycosphingolipid biosynthesis - ganglio series	3/173	0.033042484	3	0	3
dme00330	Arginine and proline metabolism	5/173	0.033352495	5	0	5
dme04142	Lysosome	12/173	0.048966513	12	6	6
dme00052	Galactose metabolism	4/173	0.055359757	4	0	4
				=		
dme04145	Phagosome	9/173	0.057041593	9	3	6
dme00510	N-Glycan biosynthesis	6/173	0.05920285	6	1	5

			2			
dme00220	Arginine biosynthesis	3/173	0.09377777	3	0	3

(b)

KEGGID	Description	GeneRatio	pvalue	Count	Up	Down
dme04142	Lysosome	7/57	0.005489661	7	5	2
dme04330	Notch signaling pathway	3/57	0.016363102	3	3	0
dme04310	Wnt signaling pathway	5/57	0.027364129	5	5	0
dme00513	Various types of N-glycan biosynthesis	4/57	0.047322795	4	2	2
dme00510	N-Glycan biosynthesis	3/57	0.064896658	3	1	2
dme00562	Inositol phosphate metabolism	3/57	0.071897994	3	3	0
dme00531	Glycosaminoglycan degradation	2/57	0.071989278	2	2	0
dme04144	Endocytosis	6/57	0.076377453	6	1	5
dme04391	Hippo signaling pathway - fly	3/57	0.10305646	3	0	3
dme00520	Amino sugar and nucleotide sugar metabolism	3/57	0.115930753	3	1	2
dme04213	Longevity regulating pathway - multiple species	3/57	0.120353298	3	1	2
dme04141	Protein processing in endoplasmic reticulum	5/57	0.123717515	5	0	5
dme04150	mTOR signaling pathway	4/57	0.131618237	4	4	0
dme03022	Basal transcription factors	2/57	0.136191321	2	0	2
dme00051	Fructose and mannose metabolism	2/57	0.150224069	2	2	0
dme04137	Mitophagy - animal	2/57	0.150224069	2	0	2
dme01230	Biosynthesis of amino acids	3/57	0.162772171	3	2	1
dme04320	Dorso-ventral axis formation	2/57	0.186434249	2	1	1
dme04145	Phagosome	3/57	0.219684304	3	2	1
dme00730	Thiamine metabolism	1/57	0.234171119	1	0	1

(c)

KEGGID	Description	GeneRatio	pvalue	Count	Up	Down
dme01212	Fatty acid metabolism	12/127	1.44E-06	12	6	6
dme00061	Fatty acid biosynthesis	6/127	2.63E-05	6	4	2
dme00910	Nitrogen metabolism	4/127	0.002030445	4	1	3
dme00071	Fatty acid degradation	7/127	0.002275443	7	2	5
dme00010	Glycolysis / Gluconeogenesis	8/127	0.002737712	8	3	5
dme04142	Lysosome	12/127	0.007154105	12	11	1
dme01230	Biosynthesis of amino acids	8/127	0.008529766	8	2	6
dme00270	Cysteine and methionine metabolism	5/127	0.009775389	5	1	4
dme00982	Drug metabolism - cytochrome P450	4/127	0.01285374	4	1	3
dme00040	Pentose and glucuronate interconversions	5/127	0.014620276	5	2	3
dme00410	beta-Alanine metabolism	5/127	0.014620276	5	0	5
dme00980	Metabolism of xenobiotics by cytochrome P450	4/127	0.020568699	4	1	3
dme00053	Ascorbate and aldarate metabolism	3/127	0.022447763	3	1	2
dme04215	Apoptosis - multiple species	4/127	0.02365544	4	0	4
dme01040	Biosynthesis of unsaturated fatty acids	3/127	0.027156265	3	1	2
dme00062	Fatty acid elongation	3/127	0.032349409	3	1	2
dme00280	Valine, leucine and isoleucine degradation	5/127	0.041540671	5	0	5
dme00220	Arginine biosynthesis	3/127	0.044167946	3	0	3
dme04146	Peroxisome	7/127	0.04630464	7	2	5
dme00620	Pyruvate metabolism	4/127	0.047884142	4	2	2

Table S9. Information of the 12 immune-related genes.

Gene ID	Log2FC	Gene abbreviation	Function annotation	up/down	P-value	GO terms
125030481	-4.12745035	*Muc-2*	mucin-2-like	down	7.20E-05	chitin binding
125044130	-21.19378191	*COL4α5*	collagen alpha-5(IV) chain-like && -	down	3.07191E-10	chitin binding
125036747	3.046677279	*PT-1*	peritrophin-1-like	up	0.004873681	chitin binding
125029620	-2.673152066	*Muc-4*	mucin-4-like	down	0.005000334	chitin binding
125033980	-3.501919253	*CD109*	CD109 antigen-like	down	0.003447051	endopeptidase inhibitor activity
125025743	-5.293653402	*Lys*	lysozyme-	down	0.000959673	lysozyme activity
125042957	2.032384317	*pCTS-L*	procathepsin L-like	up	0.001570572	Lysosome
125044916	2.452899721	*PLA2G15*	phospholipase A2 group XV-like	up	0.002702013	Lysosome
125036216	2.284296757	*CTSB*	cathepsin B-like	up	0.003751811	Lysosome
125039192	-2.424975478	*Casp1*	caspase-1-like	down	0.005966233	Apoptosis - fly||Apoptosis - multiple species
125046908	-11.9350385	*PO*	peroxidase-like	down	0.002251844	antioxidant activity
125030627	1.624504306	*TNFAIP8*	tumor necrosis factor alpha-induced protein 8-like protein	up	0.00308475	apoptotic process||cell death
